# Cases of Hooping-Cough

**Published:** 1823-03

**Authors:** John Webster

**Affiliations:** Licentiate of the Royal College of Physicians of London, Physician to the Royal Infirmary for Sick Children, &c.


ART. IV.—*Cases of Hooping-Cough.* By JOHN WEBSTER, M.D. Licentiate of the Royal College of Physicians of London, Physician to the Royal Infirmary for Sick Children, &c. [illegible]

IN a former Number of this Journal, a few hints were thrown out with a view to direct the attention of medical men more particularly to that affection of the head which is so commonly met with in hooping-cough. An opinion was also expressed, that it might perhaps be primary, and not secondary. But, as greater experience, and more numerous post-mortum examinations than have yet come under my observation, are necessary to be able properly to investigate, and to clear up the doubt which exists, at least in my mind, on this point, it is not meant at present further to prosecute the inquiry. However, in confirmation of what was then said of the treatment, it is thought that the following cases will be read with some interest, as they show the marked benefit of the practice recommended. The *ratio medendi* in all being to remove this affection of the head; whether primary or secondary, it remains for future experience and investigation fully to establish.

But, before proceeding to a detail of the cases, it may not be uninteresting nor out of place to observe, that pertussis cannot be classed among the diseases which attack a person only once during life. Such an idea, if any now entertain it, is altogether erroneous, notwithstanding it is supported by the high authority of Dr. Cullen, thus expressed in the 1403d section of his Practice of Physic: "This contagion, like several others, affects persons but once in the course of their lives." Experience has shown the fallacy of this remark, as instances are on record where the disease has returned oftener than once, and it has been said even more frequently. The fact is so well established, that there is not the smallest doubt on the subject; although it is at the same time acknowledged that, if an individual has once had hooping-cough, he is not very likely again to be affected with it.

CASE I.—Caroline Harvey, æt. two years and two months; 28th November, 1822. Has been ill two months. The mother says, she first complained of heaviness and pain of the head, with slight fever in the evening; the eyes were watery; and the child did not sleep well. These symptoms continued about fourteen days, when the cough and hooping first began. About a fortnight ago, complained of pain in the chest and difficulty of breathing, which have continued almost ever since. The cough and hooping have been very severe of late; and she has been very light-headed, and often delirious at night. Seldom sleeps well; has lost appetite and flesh; never has bled at the nose or mouth; and bowels are for the most part naturally opened. Has lately had from eight to ten fits of hooping in the day-time, and from thirty-five to forty during the night, particularly towards morning, when she has frequently from three to five fits in succession, which exhausts her very much. The affection of the head appears to have increased since a week or two ago. Tongue white and papular; thirst; no appetite; and she is often very sick. Has used a great deal of medicine, (quack as well as others,) but without the slightest benefit whatever: according to the mother's statement, the child got worse, instead of better, after using them. The mother cannot account for the appearance of the disease, as the patient has not, after the most strict inquiry, been exposed to catch it from any other child; and a younger sister, who never had the complaint, but who has been constantly in the same room, and playing with her, has never yet been attacked with hooping-cough. Applicentur Hirudines duæ fronti. ℞. Calomel, gr. ss.; Pulv. Rhei, gr. ijss.; Pulv. Ipecacuanhæ, gr. ss. M. ter die sumendus.—30th. Leeches bled very well; scarcely any hooping, but cough no better; breathes easier; complains less of the head; bowels open, motions very dark and offensive; is feverish at night; tongue cleaner; no appetite, still thirsty: appears more lively. Pergat. *c.* Pulv. u.a. *c.* Magnes. gr. j. loco Calomel. ℞. Antimonii Tartarizati, gr. j.; Theriacæ, Oxymellis Scillæ, āā ʒiij.; Aquæ puræ, ℥iv. fiat mist. coch. min. duo 2dis horis sumd.—4th Dec. No hooping, very slight cough, quite well, discharged.—22d. Has remained free from the disease since discharge.

Case II.—Harriet Harris, æt. four years; 30th November, 1822. Has been affected with hooping-cough for three weeks; has from five to six fits during the day, and about twelve in the night; coughs up blood, red and frothy; tongue white; bowels costive; belly swelled.—℞. Calomel, gr. j.; Pulv. Rhei, gr. iijss.; Pulv. Scillæ, gr. ½. M. ter die. Habeat balneum tepidum, o. n.—3d Dec. Hooping not any better, had twelve fits during last night; still spits blood; complains of pain of stomach; bowels open. Applicentur Hirudines duæ fronti. Pergat. *c.* Pulv. u.a. *c.* Magnesiæ gr. j. loco Calomel.—7th. Has had six fits of hooping since yesterday. An eruption, somewhat like measles, appeared on the skin this morning; bowels costive, motions dark and knotty; tongue dry and furred; thirst; urine red coloured and plentiful. ℞. Pulv. Jalapæ, gr. iijss.; Potassæ Supertartritis, gr. vjss. M. mane et vesp. sumendus. ℞. Infusi Rosæ, ℥ss.; Magnes. Sulphatis, ʒss. M. ter die. Repetatur balneum tepidum mane et vespere.—10th. Scarcely hoops any; head and skin feel very hot; eyes appear hydrocephalic; occasional delirium; pulse 140; bowels costive, motions black, knotty, and offensive; urine high coloured and scanty; screams frequently, and is always very restless. ℞. Calomel, gr. ss; Pulv. Rhei, gr. ijss.; Pulv. fol. Digitalis, gr. ¼. M. 4 ter in die sumendus. Pergat. *c.* haust. u.a. add. Spirit. Ætheris Nitrici, gt. xx. Repetantur balnea u.a.—12th. Hooping not so severe nor frequent; pulse 140; skin hot; does not scream so frequently; bowels open; eyes more clear; eruption still apparent. Applicentur Emplast. lyttæ nuchæ. Pergat. cum medicamentis et balneis u.a.—14th. Eruption quite gone; hoops oftener than before,—twelve times since yesterday; fever less violent; otherwise much better. Applicentur Hirudines duæ fronti. Pergat. *c.* haust. u.a. inter. alia.—17th. Leech-bites bled very well; has hooped only six times since the 14th; head less painful, not so drowsy; bowels open; scarcely any fever; picks her nose frequently. Pergat. *c.* mistura u.a.—19th. Hooping and cough quite gone. Inter. med.—24th. No complaint.

Case III.—Edward Harris, æt. one year and two months, (brother of the preceding patient;) 12th December, 1822. Has been ill of hooping-cough three weeks. Hoops generally three times in the day, and about six times during the night; eyes appear heavy, full of tears, and slightly inflamed; bowels costive; is feverish and thirsty,

Bled at the nose about a week ago. Applicentur Hirudines duæ fronti. ℞. Calomel, gr. ss.; Pulv. Rhei, gr. ijss.; Pulv. Ipecacuanhæ, gr. ss. M. ter die sumendus. —14th. Leeches bled freely; has not hooped any since yesterday morning; appears more lively, and eyes are less inflamed; has a considerable discharge of matter from the ears; bowels regular. ℞. Infus. Rosæ, ℥iv.; Magnes. Sulphatis, ʒiv.; Oxymellis Scillæ, ʒvj. M. coch. mod. ter die sumendus.—17th. Has not hooped at all since the 13th; only a slight cough remains; no fever; bowels open; discharge from the ears still continues. Pergat. cum mistura u.a. —22d. No return of the disease. Well.

Case IV.—Sarah Bewick, æt. six years; 12th December, 1822. Has been ill of hooping-cough eight weeks. Has about twenty-four fits during the day, and hoops almost constantly at night; brings up blood with phlegm during the attacks of her complaint, and passed some blood by stool last night. Bowels, formerly costive, now open by medicine. Complains of pain of the head; is always very drowsy, and inclined to sleep, and is very feverish at night. Is of a full florid habit of body, and is very stout for her age. Applicentur Hirudines tres fronti. ℞. Calomel, gr. j.; Pulv. Rhei, gr. iij.; Pulv. Ipecac. gr. ss. M. ter die sumendus.—14th. Leeches bled well; has less pain of head, and is not so drowsy; hooping and cough as before, and as frequent; she is often sick; tongue white; bowels open; thirst and fever diminished; complains of oppression at the chest; has not passed any more blood since admission. ℞. Oxymellis Scillæ, ʒiv.; Tincturæ Digitalis, gtt. xvj.; Aquæ puræ, ℥iv. fiat mistura cujus cochleare 4tis horis sumendum. ℞. Magnesiæ, gr. ij.; Pulv. Rhei, gr. iv. M. mane et vespere.—16th. General health improved; hooped twenty times yesterday, from six A.M. to six P.M., but the fits were not so severe or loud as before; not so sick, though she still vomits; bowels open; appetite better; breathing much easier, but has still fever at night. Applicentur Hirudines duæ fronti. Repetatur Pulv. ut 12ma. ter die. Int. mistura. —19th. Leeches bled well; cough continues bad at night, but it is not always accompanied with a fit of hooping, as was constantly the case before; hooped sixteen times yesterday, from six A.M. to six P.M., and much oftener at night; no head-ache or drowsiness; tongue clean; bowels open; breathing natural, and feels no oppression at the chest; appetite improved, and feels much better in every respect. ℞. Infusi Rosæ, ℥iv.: Oxymellis Scillæ, ʒiij.; Magnes. Sulphatis, ʒiv. M. fiat mist. cujus cochleare 4tis horis sumendum.—21st. Hooped yesterday eighteen times, from six A.M. to six P.M., and coughed seven times; bowels open; is quite lively in appearance, and sleeps much better than formerly, being less frequently disturbed with the hooping at night: no other complaint. Applicentur Hirudines duæ fronti. Pergat. cum mistura u.a.—24th. Only one leech bit, which bled very little. Hooping no better, and cough is very troublesome; vomits frequently. Applicentur Hirudines duæ pone aures. ℞. Calomel, gr. j.; Pulv. Rhei, gr. iv.; Pulv. Ipecacuanhæ, gr. ss. M. bis die sumendus.—26th, 1 P.M. Leeches bled profusely; has only had twelve fits of hooping since yesterday morning at six till now; cough much better; no fever; quite well in other respects. Applicentur Hirudo fronti. Pergat. cum pulvere u.a.—28th. Leech bit well, and bled freely: no hooping whatever since; is quite free from complaint. Intermittantur medicamenta.—31st. Discharged cured.—10th January, 1823. Continues well.

CASE V.—James Anderson, æt. one year and eight months; 12th December, 1822. Has been ill of hooping-cough seven days. Hoops about twelve times during the day, and very often at night; eyes watery, and he appears drowsy; head feels heavy to the mother; great difficulty of breathing, and is oppressed at the chest; bowels very costive, and is very feverish at night. Applicentur Hirudines duæ fronti. ℞. Calomel, gr. j.; Pulv. Rhei, gr. ijss.; Pulv. Scillæ, gr. $\frac{1}{4}$. M. ter die sumendus.—14th. Leeches were, by mistake, applied on the temples, but they bled very well; has great difficulty of breathing, and suffers much from oppression at the chest; coughs very frequently, but has not hooped above four times since yesterday morning till now. Applicentur Emplast. lyttæ sterno. ℞. Infusi Rosæ, ℥iv.; Oxymellis Scillæ, ʒv.; Magnesiæ Sulphatis, ʒiv. Fiat mistura, cujus cochleare modicum 6tis horis sumendum. Pergat. pulv. *c.* u.a. *c.* Magn. gr. j. loco Calomel.—17th. Cough still very troublesome; has only hooped three times since yesterday; head much better, and eyes less red and watery; pectoral symptoms better, but fever considerable; bowels open. Applicentur Hirudo fronti. Pergat. cum mistura u.a. ℞. Potassæ Nitratis, Pulv. Rhei, āā gr. ijss.; Pulv. Ipecacuanhæ, gr. ss. M. bis die sumendus.—25th. Leech bled well on the 17th; scarcely hooped any afterwards, and none since the 21st instant. Cough almost gone; bowels open; otherwise well. Inter. medicamenta.—27th. No complaint; discharged.

CASE VI.—Thomas Cole, æt. seven years; 17th December, 1822. Had hooping-cough last summer, for upwards of three months; at the end of which period (in August,) he became a patient at the Infirmary, the disease being still very violent and frequent. Three leeches were ordered to be applied to the forehead, which bled profusely, and a purging medicine was given every night. The hooping ceased almost immediately after the evacuation of the blood, nor did it ever again return; and in less than a fortnight he was discharged free from complaint.

Has remained quite well ever since, till about a week ago, when he began to complain of pain in the head and frequent cough, but he has not been observed to hoop until within these three days. The fits have been during that time most violent, recurring at least every quarter of an hour, as well in the day as during the night; he has complained also of constant pain in the head and forehead. He gets very blue in the face after a fit, and is, by the mother's account, frequently convulsed. Tongue white; bowels open; vomits frequently, and is very restless and feverish at night. Has bled freely at the nose last night, and this morning appears better, though the hooping still remains as frequent and severe as before. Applicentur Hirudines quatuor fronti. —℞. Calomel. gr. ss.; Pulv. Rhei, gr. ijss.; Pulv. Scillæ, gr. $\frac{1}{4}$. M. ter die sumendus.—19th. Leeches bled profusely; has not hooped any since; head feels relieved; eyes very watery and swelled. An eruption appeared on the skin this morning, distinct, but not the measles; cough continues very frequent, and he is feverish at night. ℞. Infusi Rosæ, ℥iv.; Oxymellis Scillæ, ʒvj.; Magnes. Sulphatis, ʒiv. M. cochl. mod. 4tis horis sumend.—21st. No hooping; eruption gone; fever not so violent; cough troublesome; bowels open; thirst; complains of pain of head, and feels always very heavy, and inclined to sleep; appetite moderate. Applicentur Emplast. lyttæ nuchæ. Pergat. cum mist. add. Tinct. Digitalis, ℈ij.—24th. Cough better; complains of constant pain of the right side of the head; breathing a little difficult, and accompanied with wheezing, but feels no pain in the chest; sleeps much better, and eyes are more clear; slightly feverish at night, and is very weak. Pergat. cum mistura add. Vini Ipecacuanhæ, ℈ij. loco Tinct. Digitalis.—26th. Cough very frequent; pain of head as before, but his spirits are better, and the appetite improves. Pergat. cum mist. —28th. Cough very troublesome; pain of head better; slight accession of fever in the evening, and feels weak and exhausted. ℞. Pulv. Cinchonæ, Sodæ Carbonatis, āā gr. iij. M. ter die sumendus. ℞. Mist. Theriacæ, ʒij. pro re nata: inter. alia.—2d January, 1823. Scarcely any complaint; improves daily. Pergat. cum medicamentis, u.a.—6th. Convalescent; discharged.

Case VII.—Charles Forrest, æt. one year and four months; 24th December, 1822. Ill about a week, with frequent cough; has hooped, for the last four days, about five times in twenty-four hours. Had lately a discharge of matter from both ears, and is affected with a sore at the angle of the mouth. Discharge from the ears has ceased a few days ago, since which he has begun to hoop: had two fits of hooping yesterday, and three in the night. He appears heavy about the eyes and head; breathes easy, and expectorates very little: is thirsty and feverish; bowels costive; never bled any at the nose. The mother cannot account for the origin of the disease, not being aware of her child's having been ever exposed to infection. Applicentur Hirudines duæ fronti. ℞. Calomel, gr. ss.; Pulv. Rhei, gr. ijss.; Pulv. Ipecac. gr. ¼. M. ter die sumendus.—26th. Leeches bled very well; no hooping whatever since; cough still very frequent; expectorates phlegm; breathes easy; bowels open; urine red and plentiful; passes restless nights, and sleeps ill. ℞. Infusi Rosæ, ℥iv.: Magnes. Sulphatis, ʒvj.; Oxymellis Scillæ, ʒvj. M. cochleare modicum 4tis horis.—31st. Hooping again returned on the 29th: has now a fit of the disease at least every hour; bowels open; is not feverish. Applicentur Hirudines duæ fronti. Pergat. cum mistura u.a.—4th January, 1823. Leeches bled profusely; has only hooped three times since: the last fit on the 1st instant; cough frequent; is much troubled with a copious discharge of mucus from the nose; no fever; bowels costive. Pergat cum mistura, et repetantur pulv. ter die, ut 24th ult.—7th. No hooping whatever since the 1st instant. Convalescent; discharged.

Case VIII.—Elizabeth Ryner, æt. four months; 31st December, 1822. Ill a month; has generally about six fits of hooping, sometimes very violent, in twenty-four hours; gets blue in the face after them; eyes hydrocephalic; bleeds at the mouth; cough very severe; bowels natural; tongue white; feverish at night, and vomits frequently. Has not used any medicine. Applicentur Hirudines duæ fronti. ℞. Cal. gr. ss.; Pulv. Rhei, gr. ij.; Pulv. Ipecacuanhæ, gr. $\frac{1}{3}$. M. ter die sumendus.—4th January, 1823. Leeches bled very well; has only hooped once since, (on the 2d instant;) coughs frequently; breathes easier, and still expectorates phlegm; less fever; bowels open four times a-day; is always very sleepy, but the eyes are now quite shut during sleep, and more clear and natural in appearance. ℞. Magnes. gr. j.: Pulv. Rhei, gr. ijss.; Pulv. Scillæ, gr. $\frac{1}{5}$. M. ter die sumendus, inter. alia.—7th. Scarcely any complaint, except a slight cough. ℞. Mist. Oxymellis Scillæ, ʒij. pro re nata.—10th. Cured; discharged.

Case IX.—Abigail Hancock, æt. nine years; 31st December, 1822. Ill of hooping-cough about fourteen days: hoops generally twelve times in the day, but not so often at night; complains of pain over the eyes and in the forehead; feels much difficulty of breathing, and has frequent cough; pulse 106; tongue very much furred; bowels open only by medicine; has lately bled at the nose. For a week previous to the appearance of the disease, the mother said the child complained of headache, and appeared very heavy about the eyes. Applicentur Hirudines quatuor fronti. ℞. Calomel, gr. j.; Pulv. Rhei, gr. iij.; Pulv. Scillæ, gr. $\frac{1}{2}$. M. ter die sumendus.—2d January, 1823. Leeches bit well, and bled profusely; has not hooped any since; no pain of head: complains still of difficulty of breathing, and of pain in the chest, but less so than before; tongue white; bowels open; coughs frequently. ℞. Infusi Rosæ, ℥iv.; Oxymellis Scillæ, ʒvj.; Magnes. Sulphatis, ʒiv. M. coch. 4ter. in die sumenda. ℞. Potassæ Nitratis, gr. iij.; Pulv. Rhei, gr. iv.; Pulv. Scillæ, gr. $\frac{1}{2}$. M. bis die sumendus, inter alia.—4th. Scarcely any complaint, except difficulty of breathing and slight cough, with very little fever; bowels open; tongue white. A vesicular eruption appeared this morning on the chest; pulse 100. Pergat. cum medicamentis u.a.—7th. No hooping; very weak; rather more fever, which is now preceded by a little shivering in the evening. ℞. Pulv. Cinchonæ, Sodæ Carbonatis, āā gr. iij. M. ter die sumendus. ℞. Spiritus Ætheris Nitrici, gtt. xij. ex aqua et sacharo 4ter in die sumendas, inter. alia. —9th. Improves daily, scarcely any complaint; gets strength; eruption gone. Pergat. cum pulv. et guttis, u. a.—10th. Quite well: discharged.

Case X.—Elizabeth Stratton, æt. one year and six months; 4th January, 1823. Ill six weeks: hoops at least once every hour in the day, as well as in the night; face frequently becomes blue and swelled after a fit, and the eyes appear very heavy; has scarcely any difficulty of breathing, except towards night; bowels open; restless and feverish at night; has never bled any at the nose or mouth. Appl. Hirudines duæ fronti. Nihil præscribendum est.—14th. Leeches bled profusely; has never hooped since their application: all other complaints soon left the patient, and she is now quite well. As the child improved so rapidly, the mother did not think it neçessary to return to the Infirmary till to-day. Discharged.

Case XI.—Communicated by Dr. Gregory: it is considered of importance, seeing that the benefit which followed the treatment was as decided in his hands as in mine.

Anne Maycer, æt. six years; 2d December, 1822. Ill four weeks, but has hooped only during the last three. Has coughed very violently, both night and day, the last eight days, and brings up phlegm, but not her victuals. Pain on coughing referred to the larynx. Has rapidly emaciated; cannot be sent to school, and seldom sleeps. Her brother and sister have both hooping-cough at present. ℞. Lotion: Antim. Tartar. cum Tinct. lyttæ, pectori, mane et vespere utendam. ℞. Calomel, gr. ij.; Pulv. Scammoniæ, gr. vj. M. o. n. ℞. Extracti Conii, gr. iij.; Vini Ipecac. gtt. x.; Magnes. Vitriol. ℈j.; Aquæ, ℥j. M. ter die capiat.—4th. Decided pertussis, and very severe; has great pain of the head; is much emaciated. Fits last about half an hour. Applicentur Hirudines iv. fronti. ℞. Pulv. Scammoniæ, gr. vj.; Calomel, gr. ij.; o. n. ℞. Ungt. Antim. Tartar. pectori, mane et vespere.—6th. Does not hoop near so often as before, about six times the last twenty-four hours; her spirits are much better; slept pretty well; but is quite afraid of the cough; head better; motions natural. Persev. cum pulvere, o.n. u.a. ℞. Plumbi Superacetatis, gr. j; Tinct. Digitalis, ʒj; Aquæ, ℥iij. M. sumat ℥ss. 6tis horis.—9th. By account, better: skin has come out in an eruption. Cont. cum mistura u.a. et rept. pulvis, o.n. u.a.—11th. Hooping and cough better; less pain of the head, eyes clear; bowels open. Repetantur Hirudines ij. fronti. ℞. Calomel, gr. ij.; Pulv. Rhei, gr. iv.; Pulv. Antimonialis, gr. ijss. M. o.n. sumat. Omitt. alia.—14th. Severe pain of head; and she hoops much oftener now than she did three days ago; twelve times last night, and more violently: sleeps well; eyes look heavy; eruption gone. Leeches ordered on the 11th did not bite at all. Repetantur Hirudines ij. fronti. Pergat. cum mistura ut December 6, inter. alia.—16th. These leeches bit very well: decidedly better to-day, and has hooped only twice yesterday; since the leeches fell off, has made much water. Pergat. cum Calomel et Scammoniæ, gr. v. o.n.—18th. *No hooping;* cough much better; no pain of head; sleeps well; begins to run about with the other children. Cont. cum pulvere o. n. Omitt. mistura.—23d. Only very slight cough, about twice a-day; gets flesh and colour; quite well. Omitt. medicamenta.—27th. No return of disease. Cured.

Many other examples, illustrative of the same kind of practice, might be quoted from the register kept at the Infirmary; but it is superfluous, and would only be tiresome to the reader, to do so. In regard to the success attending it, there can be no doubt, for experience fully justifies the observation that it is most decided; and though instances may occur, either from the long continuance of the disease, and the consequent exhaustion, organic derangement, or other unusual causes, which may prevent the adoption of the plan now spoken of, or render it abortive when tried,—nevertheless these exceptions do not militate against the general rule, nor destroy the well-founded prospect of benefit which in most cases may be expected, with confidence, to follow the mode of treatment recommended.

*10th January,* 1823.

